# SARS-CoV-2 Omicron Subvariants Do Not Differ Much in Binding Affinity to Human ACE2: A Molecular Dynamics Study

**DOI:** 10.1021/acs.jpcb.3c06270

**Published:** 2024-04-02

**Authors:** Hoang Linh Nguyen, Thai Quoc Nguyen, Mai Suan Li

**Affiliations:** † Institute of Fundamental and Applied Sciences, Duy Tan University, Ho Chi Minh City 700000, Vietnam; ‡ Faculty of Environmental and Natural Sciences, Duy Tan University, Da Nang 550000, Vietnam; § Faculty of Physics, VNU University of Science, Vietnam National University, 334 Nguyen Trai, Hanoi 100000, Vietnam; ∥ Dong Thap University, 783 Pham Huu Lau Street, Ward 6, Cao Lanh City, Dong Thap 81000, Vietnam; ⊥ Institute of Physics, Polish Academy of Sciences, al. Lotnikow 32/46, Warsaw 02-668, Poland

## Abstract

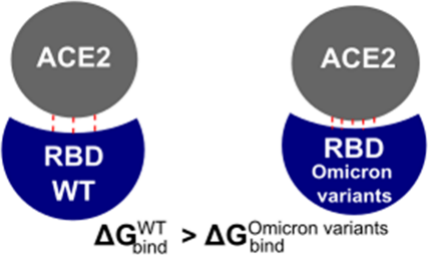

The emergence of the variant of concern Omicron (B.1.1.529) of severe acute respiratory syndrome coronavirus 2 (SARS-CoV-2) exacerbates the COVID-19 pandemic due to its high contagious ability. Studies have shown that the Omicron binds human ACE2 more strongly than the wild type. The prevalence of Omicron in new cases of COVID-19 promotes novel lineages with improved receptor binding affinity and immune evasion. To shed light on this open problem, in this work, we investigated the binding free energy of the receptor binding domain of the Omicron lineages BA.2, BA.2.3.20, BA.3, BA4/BA5, BA.2.75, BA.2.75.2, BA.4.6, XBB.1, XBB.1.5, BJ.1, BN.1, BQ.1.1, and CH.1.1 to human ACE2 using all-atom molecular dynamics simulation and the molecular mechanics Poisson–Boltzmann surface area method. The results show that these lineages have increased binding affinity compared to the BA.1 lineage, and BA.2.75 and BA.2.75.2 subvariants bind ACE2 more strongly than others. However, in general, the binding affinities of the Omicron lineages do not differ significantly from each other. The electrostatic force dominates over the van der Waals force in the interaction between Omicron lineages and human cells. Based on our results, we argue that viral evolution does not further improve the affinity of SARS-CoV-2 for ACE2 but may increase immune evasion.

## Introduction

The outbreak of severe acute respiratory syndrome coronavirus 2 (SARS-CoV-2)^1^ caused one of the most devastating pandemics (COVID-19) in human history. As of February 2023, COVID-19 has claimed about 6.8 million lives and over 672 million confirmed cases worldwide.^2^ SARS-CoV-2 is a new member of the beta genera of coronavirus related to SARS-CoV-1 and MERS-CoV.^1^ The virion has a spherical shape, with a spike (S) protein protruding from the surface. The S protein plays an important role in viral infection as it binds to host cells during the first step of virus invasion, which makes it a popular target for antibody and vaccine design.^3−7^ The anchor of the receptor binding domain (RBD) of the S protein to the human Angiotensin-converting enzyme 2 (ACE2) initiates the entry of the virus into the host cell^8,9^ (the RBD actually binds to the peptidase domain of ACE2, but for simplicity, we say that it binds to ACE2). The increased efficiency of SARS-CoV-2 binding to ACE2 compared to SARS-CoV-1^10^ probably contributes to the higher infectivity of this virus. Therefore, there have been many experimental^10,11^ and computational^12−15^ work on the mechanism of RBD-ACE2 interaction.

In November 2021, a variant of concern Omicron (B.1.1.529) appeared causing the fourth wave of the pandemic.^16^ Although the virulence of Omicron is lower than that of the Delta variant,^17,18^ its transmissibility and immune evasion are higher compared to the Delta variant.^19^ The high infection rate of Omicron promotes the evolution of the virus to improve receptor binding fitness as well as antibody and vaccine evasion.^20^ As a result, many Omicron subvariants emerged and spread across countries.^21^ Experimental studies have found that Omicron subvariants alter the binding affinity of the S protein for ACE2 and antibody evasion (Table 1).^22,23^ Here the binding affinity is characterized by the dissociation constant *K*_D_ or the binding free energy Δ*G*_bind_, so that the lower the *K*_D_/Δ*G*_bind_, the higher the binding affinity. A mutation that increases the binding affinity for ACE2 can also increase the transmissibility of the virus.^24,25^ Thus, understanding the molecular mechanism behind the change in S protein binding to host cells should help prepare for a surge of novel variants.

**Table 1:** Mutations of Omicron Sub-variants Studied This Work and Experimental *K*_D_ (nM) of the Binding of RBD/Spike Protein and ACE2^a^

no.	subvariant	*K*_D_ (nM)	experimental binding energy (kcal/mol)	average of experimental binding energy (kcal/mol)	MM-PBSA binding free energy (kcal/mol)
1	BA.1	19.5,^26^ 20.8,^73^ 14.5,^74^ 7.8,^75^ 19.7 ± 1.1,^76^ 36.94 ± 4.58,^76^ 25.3 ± 1.2,^77^ 8.85^19^	–10.58, −10.55, −10.76, −11.13, −10.58 ± 0.03, −10.20 ± 0.07, −10.43 ± 0.03, −11.05	–10.66 ± 0.29	–27.97 ± 2.91^35^
2	BA.2	10.0,^26^ 9.4,^71^ 35.3,^73^ 4.0,^78^ 0.95,^79^ 10.8,^74^ 4.0,^75^ 23.9 ± 2.1,^76^ 44.96 ± 3.23,^76^ 1.19,^80^ 0.95,^79^ 1.49 ± 0.05^81^	–10.98, −11.02, −10.23, −11.53, −12.39, −10.94, −11.53, −10.46 ± 0.05, −10.09 ± 0.04, −12.25, −12.39, −12.12 ± 0.02	–11.33 ± 0.79	–33.42 ± 3.61
3	BA.2.3.20				–34.72 ± 1.30
4	BA.2.75	1.8,^82^ 2.2,^71^ 0.45,^78^ 0.17^80^	–12.00, −11.88, −12.83, −13.41	–12.53 ± 0.62	–39.36 ± 1.88
5	BA.2.75.2				–39.25 ± 3.56
6	BA.3	22.1,^26^ 26.5^74^	–10.51, −10.40	–10.45 ± 0.05	–28.37 ± 1.32
7	BA.4/BA.5	13.3,^71^ 6.0,^78^ 0.61,^79^ 14.4,^74^ 2.4,^75^ 14.4 ± 0.3,^76^ 28.06 ± 3.15,^76^ 0.4,^80^ 1.08 ± 0.16^83^	–10.81, −11.29, −12.65, −10.76, −11.83, −10.76 ± 0.01, −10.37 ± 0.07, −12.90, −12.31 ± 0.09	–11.52 ± 0.88	–36.26 ± 2.62
8	BA.4.6				–35.70 ± 2.12
9	BJ.1				–36.53 ± 1.84
10	BN.1				–38.69 ± 3.89
11	BQ.1.1	8.1,^82^ 0.56,^79^ 0.66 ± 0.11^83^	–11.11, −12.70, −12.60 ± 0.10	–12.14 ± 0.73	–36.85 ± 3.08
12	CH.1.1				–36.26 ± 0.42
13	XBB/XBB.1	19.0,^82^ 2.06,^79^ 1.00 ± 0.07^81^	–10.60, −11.92, −12.35 ± 0.05	–11.26 ± 0.66	–37.33 ± 4.57
14	XBB.1.5	3.4^82^	–11.62	–11.62	–37.83 ± 3.95

a The binding free energy values are calculated using equation Δ*G*_bind_ = *RT*ln (*K*_D_), here *T* = 300 K and *R* = 0.001987204259 kcal/mol/K. The errors represent standard deviations.

The predominance of the Omicron variant facilitates the recombination of existing lineages, leading to the emergence of novel lineages. Lineage BA.2 and BA.4/BA.5 have a stronger proximity to ACE2 than BA.1.^23,26^ The sublineage BA.2.75 originated from BA.2 has enhanced infection ability and binding affinity for ACE2 in comparison with BA.2 and BA.5 lineages.^27^ The N460K and G339H mutations of BA.2.75 were found to increase the ability of BA.2.75 to bind to ACE2. Omicron sublineages such as XBB, XBB.1, XBB.1.5, CH.1.1, and BQ.1.1 became the predominant variants. Experiments indicate a difference in the binding affinity of these subvariants to ACE2.^23^ Thus, deciphering the effect of mutations in subvariants on the mechanism of binding to ACE2 can reveal hotspots in RBD, which will help us both in pharmacotherapy and in predicting future variants.

Besides experimental studies, computational tools have also been utilized to investigate complexes of ACE2 with various Omicron subvariants. The interaction between ACE2 and RBD of Omicron BA.1, BA.2, and BA.3 was analyzed using docking and molecular dynamics (MD) simulations.^28^ Using an artificial intelligence model and docking simulation, it was shown that due to a higher binding ability to the host cell, Omicron is more contagious than the WT virus.^29,30^ The artificial intelligence techniques were used to predict binding affinity changes between ACE2 and SARS-CoV-2 variants.^31^ The deep learning method was utilized to obtain the change in binding free energy caused by mutations in Omicron subvariants.^32^ Combination of MD simulation with the molecular mechanics Poisson–Boltzmann/generalized Born surface area (MM-PB/GBSA) methods can explain why Omicron BA.1 binds to ACE2 more strongly than the ancestral strain.^33−35^ Using the MM-GBSA, Singh et al. investigated the binding of ACE2 with RBD of the BA.4 and BF.7 Omicron lineages,^36^ showing that these subvariants bind to ACE2 more tightly than Omicron BA.1. Verkhivker et al. studied the effect of mutations in subvariants BA.2.75 and XBB.1.5 by molecular simulations and dynamic network models.^37^ In Lin et al. HADDOCK docking scores show the BA.2.75 and BA.5 subvariants bind to ACE2 more strongly than the wild type (WT).^38^ An *in silico* study of BA.1, BA.2, BA.3, BA.5, and BA.2.75 subvariants revealed similar behavior.^39^ The MM-PBSA method was used to estimate the binding free energy of BA.1 and BA.2 subvariants with ACE2,^40^ while the MM-GBSA method was utilized to study the interaction of BA.1, BA.1.1, BA.2, BA.3, BA.4/5,^41^ and XBB.1.5^42^ with ACE2. Emma et al. obtained the free energy of Omicron BA.2 binding with the human cell using a well-tempered extended adaptive biasing force algorithm.^43^

Thus, different groups used different computational methods to study one or more subvariants of Omicron, making it difficult to compare their results. Moreover, the subvariants of Omicron BA.2.3.20, BA.4.6, BJ.1, BN.1 XBB/XBB.1, BQ.1.1, and CH.1.1 have not been theoretically investigated. In this work, we performed MM-PBSA simulations for a relatively large number of subvariants to determine the effect of mutations in the subvariants on the receptor binding mechanism, which may disclose a trend in virus evolution. Our set consists of 14 SARS-CoV-2 sublineages, including BA.2, BA.3, BA.4/BA.5, BA.2.3.20, BA.2.75, BA.2.75.2, BA.4.6, BJ.1, BN.1 XBB/XBB.1, XBB.1.5, BQ.1.1, and CH.1.1. We have shown that among the Omicron sublineages, BA.2.75 and BA.2.75.2 bind to ACE2 more strongly than others. The binding free energies obtained from simulations have a high correlation with the experiment. The MM-PBSA method demonstrates that the electrostatic interaction between the RBD and ACE2 dominates the van der Waals interaction. However, the electrostatic interaction is not a key factor in distinguishing the binding affinities of new Omicron lineages. The mutations G339H, D405N, N440K, L452R, N460K, T478K, E484A, and Q498R of lineages increase the nonbonded interaction between RBD and ACE2 compared to WT. D405N, L452R, and N460K in new lineages enhance binding compared to that of BA.1. The mutations G339D, R346T, and K417N weaken the RBD-ACE2 interaction compared to WT, while R346T, R408S, and R493Q decrease nonbonded interaction with ACE2 compared to Omicron BA.1.

## Materials and Methods

### Models

In principle, to describe the interaction of the SARS-CoV-2 S protein with ACE2, the structure of the complete three-chain S protein should be used. However, since ACE2 interacts with the RBD in the open state, instead of the full structure, only the RBD can be considered as a reasonable approximation that is widely accepted.^44^ Therefore, in this work, simulations were carried out for RBD-ACE2 complexes.

**Figure 1:**
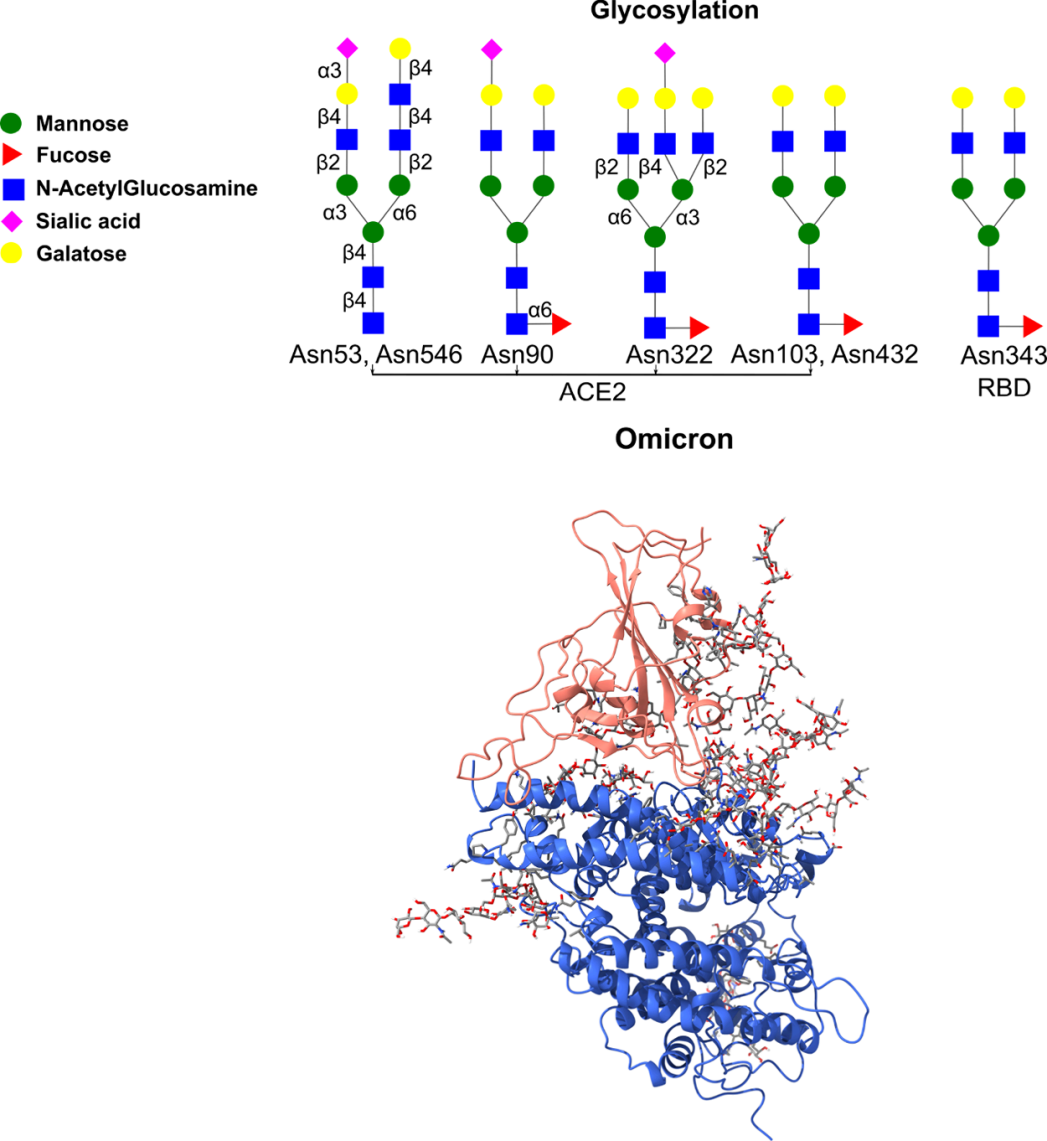
(Upper) Glycan model used in this work. (Lower) Initial complex structure of viral RBD and human ACE2 with PDB ID 7ZF7.

The RBD structures of the SARS-CoV-2 S protein in complex with the ACE2 were obtained from the Protein Data Bank (PDB) with the PDB id 7ZF7 for sublineages.^45^ The missing residues were added by the CHARMM-GUI web server.^46−49^ In general, the definition of the RBD varies slightly between groups. In our model, the RBD comprises of 334–527 residues, whereas in Lan et al. it extends between residues 333–527.^8^ A bit shorter version of the RBD with 336–518 residues was adopted by Mehdipour and Hummer^50^ Six glycans are located at residues 53, 90, 103, 322, 432, and 546 of the ACE2 and at residue 343 of the viral RBD. In this work, we used a heterogeneous setup where glycan sites have different glycans^50−52^ (Figure 1).

Sublineage mutations were generated in the 7ZF7 structure using the CHARMM-GUI web server.^46−49^ Mutations of the subvariants studied in this work are shown in Table S1 in the Supporting Information (SI). The Zn ion in the original ACE2 structure was retained. The initial structures are listed in Figure 1.

### Molecular Dynamics Simulation

The AMBER19SB and GLYCAM06j force fields were used to describe proteins and glycans.^53,54^ The systems were solvated in a rectangular box filled with 4-points OPC water molecules with a minimum distance of 1.3 nm from the solute to the edge of the box.^55^ We used the AMBER19SB force field and OPC water model because this choice has better predictive power than other options for modeling sequence-specific behavior and protein mutations.^53^ To neutralize the system, Na^+^ and Cl^–^ ions were added, maintaining the salt concentration at a physiological level of 0.15 M.

The GROMACS 2022.3 package was used for MD simulation.^56^ The solvated systems were minimized by a steep descent algorithm for structure relaxation. The system was then equilibrated in NVT and then in NPT ensembles at 300 K and 1 atm for 500 ps and 5 ns MD runs, respectively. The v-rescale and Parrinello–Rahman algorithms were used to maintain constant temperature and pressure, respectively.^57,58^ At the equilibration stage, the heavy atoms of the protein-glycan complexes were restrained by a harmonic potential with a spring constant *k* = 1000 kJ/mol/nm^2^.

To estimate the binding free energy by the MM-PBSA method, for each system, 5 independent MD simulations with a duration of 200 ns were carried out without any restraints at 300 K and 1 atm. We used a cutoff of 1.0 nm for nonbonded interactions. The PME method was used to calculate the electrostatic interaction.^59^

### MM-PBSA Method

In the MM-PBSA method, the binding free energy was calculated using the following equation:
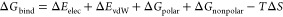
1

Here Δ*E*_elec_ and Δ*E*_vdW_ are the energies of the electrostatic and vdW interactions between RBD and ACE2. Δ*G*_polar_ is the polar solvation energy, which was calculated using Delphi software with dielectric constants for solute and solvent of 1 and 80, respectively.^60^ The nonpolar solvate energy Δ*G*_nonpolar_ = γΔ*S*ASA, where γ = 0.0072 kcal/mol/nm^2^, and SASA is solvent accessible surface area which was calculated using the gmx sasa tool in the GROMACS package with a solvent probe radius of 1.4 Å.^61^ The entropy contribution *T*Δ*S* was evaluated using the method proposed by Duan et al.^62^

### Side-Chain Contact

A side chain contact between two residues is formed when the distance between the centers of mass of their side chains is ≤6.5 Å.

## Results and Discussion

### Equilibration of RBD-ACE2 Complexes

To calculate the binding free energy of the RBD-ACE2 complexes of SARS-CoV-2 subvariants using the MM-PBSA method, 5 independent 200 ns MD runs were performed for each complex. To check whether the system has reached equilibrium, the time dependence of the mean square displacement (RMSD) with respect to the original structure was monitored. As can be seen from Figure S1, after about 100 ns, the RMSD reaches saturation, which means that the equilibration time is about 100 ns. Therefore, the first 100 ns were excluded, and the structures collected during the last 100 ns were selected to estimate Δ*G*_bind_.

### BA.2.75 and BA.2.75.2 Subvariants Bind to ACE2 more Strongly than Others

Using snapshots recorded in the last 100 ns of the MD trajectory and the MM-PBSA method (eq 1), the binding free energy of 14 SARS-CoV-2 subvariants was calculated (Table 2). Δ*G*_bind_ varies from −27.97 ± 2.91 (BA.1) to −39.36 ± 1.88 kcal/mol (BA.2.75), which is significantly lower than the experimental Δ*G*_bind_ extracted from *K*_D_. The fact that the MM-PBSA method cannot accurately predict the absolute binding free energy was previously reported.^63^ One possible reason is that our MD runs are not long enough to observe binding and unbinding events, and the entropy contribution was estimated based on the configurations around the binding site. In the case of RBD-ACE2, another possible reason for the large negative Δ*G*_bind_ value is the strong electrostatic interaction at the interface due to the large number of charged residues.^35,44^

**Table 2:** Theoretical Binding Free Energy (kcal/mol) of the Omicron Subvariants^a^

no.	subvariant	Δ*E*_elec_	Δ*E*_vdW_	Δ*G*_polar_	Δ*G*_nonpolar_	–*T*Δ*S*	Δ*G*_bind_
1	BA.1^35^	–1909.30 ± 18.80	–153.72 ± 6.09	2029.94 ± 40.79	–25.84 ± 2.49	30.94 ± 2.37	–27.97 ± 2.91
2	BA.2	–1733.89 ± 26.93	–151.99 ± 17.66	1835.73 ± 30.54	–26.15 ± 3.24	42.88 ± 4.28	–33.42 ± 3.61
3	BA.2.3.20	–1933.49 ± 60.40	–159.20 ± 13.14	2041.48 ± 71.75	–26.80 ± 2.01	41.79 ± 5.67	–34.72 ± 1.30
4	BA.2.75	–1774.89 ± 56.50	–143.79 ± 11.15	1862.27 ± 59.91	–23.17 ± 1.95	42.57 ± 5.31	–39.36 ± 1.88
5	BA.2.75.2	–1584.37 ± 32.53	–151.40 ± 21.12	1681.43 ± 48.73	–25.65 ± 2.79	40.73 ± 1.48	–39.25 ± 3.56
6	BA.3	–1991.92 ± 73.49	–145.63 ± 8.41	2094.07 ± 65.38	–23.98 ± 1.25	39.47 ± 4.43	–28.37 ± 1.32
7	BA.4/BA.5	–1836.06 ± 45.79	–141.10 ± 7.88	1922.61 ± 53.55	–23.12 ± 1.52	41.41 ± 5.70	–36.26 ± 2.62
8	BA.4.6	–1391.31 ± 48.09	–149.62 ± 15.01	1492.39 ± 62.36	–24.50 ± 2.98	40.73 ± 1.85	–35.70 ± 2.12
9	BJ.1	–1654.04 ± 50.69	–150.70 ± 19.66	1748.40 ± 72.26	–26.69 ± 3.32	46.50 ± 5.09	–36.53 ± 1.84
10	BN.1	–1771.83 ± 22.62	–138.05 ± 14.52	1857.14 ± 30.55	–22.06 ± 5.08	38.19 ± 2.44	–38.69 ± 3.90
11	BQ.1.1	–1646.12 ± 41.17	–149.13 ± 9.51	1743.44 ± 35.16	–24.94 ± 1.31	39.90 ± 5.75	–36.85 ± 3.08
12	CH.1.1	–1847.18 ± 28.28	–163.42 ± 19.28	1965.58 ± 43.63	–27.78 ± 2.31	40.62 ± 3.61	–36.26 ± 0.42
13	XBB/XBB.1	–1854.65 ± 39.66	–152.82 ± 8.19	1955.03 ± 49.32	–26.57 ± 1.41	41.68 ± 5.88	–37.33 ± 4.57
14	XBB.1.5	–1815.82 ± 60.49	–152.86 ± 24.91	1917.04 ± 78.83	–26.23 ± 3.83	40.04 ± 4.29	–37.83 ± 3.95

a Results were obtained using the MM-PBSA method, and snapshots from the last 100 ns of 5 MD runs were used for data analysis. Errors are standard deviations. Results for BA.1 are taken from ref (35).

**Figure 2:**
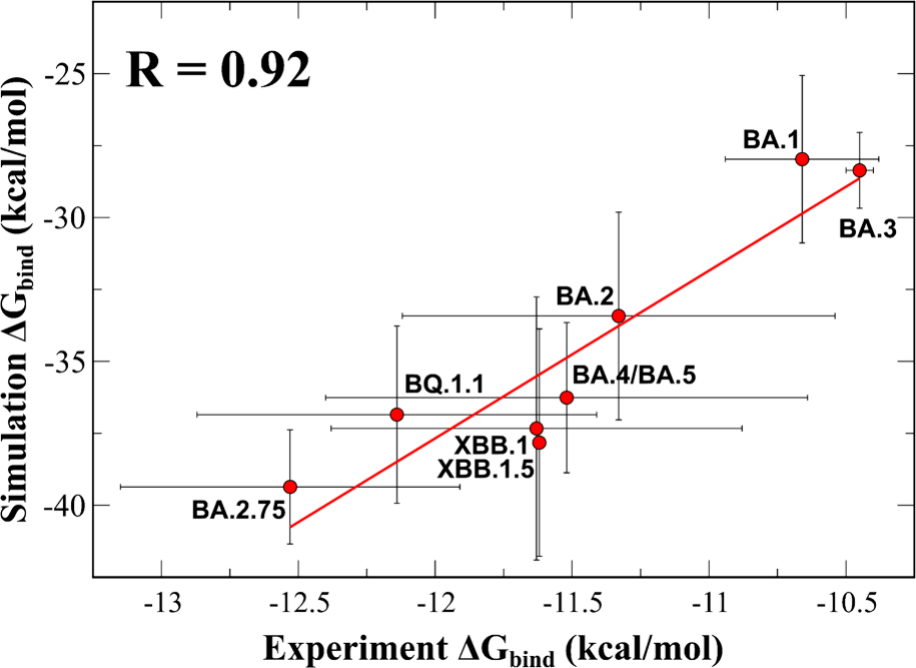
Δ*G*_bind_ obtained from MM-PBSA as a function of the experimental Δ*G*_bind_. Experimental data are only available for 8 variants. Linear fit *y*= (5.8272 ± 0.9797) × *x* + (32.2539 ± 11.2692) where *x* and *y* correspond to experimental and theoretical Δ*G*_bind_. The correlation coefficient *R* = 0.92 ± 0.06.

Although MM-PBSA cannot reproduce the absolute experimental Δ*G*_bind,_ it is useful for determining relative binding affinity as the correlation between simulation and experiment is high with a correlation level of R = 0.92 (Figure 2). From a linear fit, we get
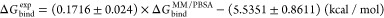
2

This equation allows us to estimate the experimental binding free energy from the simulated value.

For BA.2.3.20, BA.2.75.2, BA.4.6, BJ.1, and CH.1.1, for which there is no experimental data, using theoretical Δ*G*_bind_ from Table 2 and eq 2 we predict Δ*G*_bind_^exp^= −11.51, −12.29, −11.68, −11.82, and −11.77 kcal/mol, respectively. Therefore, these sublineages have an advantage over BA.1 (−10.66 ± 0.29 kcal/mol) in ACE2 binding.

Both Δ*G*_bind_^MM/PBSA^and Δ*G*_bind_^exp^ of BA.2.75 (Figure 2) and Δ*G*_bind_^MM/PBSA^ of BA.2.75.2 (Table 1) are lower than the other subvariants, suggesting their better binding to ACE2. However, these BA.2.75 subvariants do not predominate in new pandemic cases, indicating that the binding affinity of the spike protein for ACE2 is probably not a critical factor in the infectivity.

Within the error range, the difference in binding energy between subvariants is not significant compared to the change of binding from SARS-CoV-1 and SARS-CoV-2.^12^ Student *t* test *p*-values between SARS-CoV-2 WT and all Omicron subvariants studied in this work are less than 0.05 (Table S2, column 2), suggesting that the differences are significant. Of the 91 possible pairs between the 14 Omicron subvariants (91 = 14 × (14 – 1)/2), 59 pairs have a *p*-value greater than 0.05 (Table S2), implying that about 64% (59/91 ≈ 64%) the pairs do not differ significantly in binding affinity. The number of pairs with *p*-values >0.05 appears to increase over time (Figure S2), suggesting that evolution is reducing the variance between Omicron subvariants. We argue that this is due to two reasons. First, there is a trade-off between receptor binding and antibody escape,^64^ which leaves no room for improvement of virus receptor binding. Second, SARS-CoV-2 is not under pressure to optimize further receptor binding fitness because the current affinity (Δ*G*_bind_ ∼ −12 kcal/mol, *K*_D_ ∼ nM) is sufficient for transmission.

Since mutations N440K, N460K, and T478K play a key role in the stability of the RBD-ACE2 complex, we performed additional MD simulations for the three complexes RBD (N440K)-ACE2, RBD (N460K)-ACE2, and RBD (T478K)-ACE2. Here RBD (N440K), RBD (N460K), and RBD (T478K) refer to RBD with single mutations N440K, N460K, and T478K, respectively. Our goal is to see whether these important mutations strongly increase the binding affinity. The MD setup is similar to that in other cases. Note that N440K and T478K are present in the BA.2 variant, while XBB.1.5 contains N460K. Interaction energies of these mutations with ACE2 in the single mutation case (RBD (N440K)-ACE2, RBD (N460K)-ACE2, and RBD (T478K)-ACE2) as well as in BA.2 (N440K and T478K) and XBB.1.5 (N460K) variants are shown in Table S3. The contribution of these mutations in single point mutants is somewhat weaker than that in Omicron subvariants, presumably due to the absence of other mutations in single-point mutants that lead to a weaker interaction between RBD and ACE2. Consequently, in silico variants RBD (N440K)-ACE2 (Δ*G*_bind_ = −20.95 kcal/mol) RBD (N460K)-ACE2 (Δ*G*_bind_ = −21.78 kcal/mol) and RBD (T478K)-ACE2 (Δ*G*_bind_ = −19.96 kcal/mol) (Table S4) are not as stable as variants BA.2 (Δ*G*_bind_ = −33.42 kcal/mol) and XBB.1.5 (Δ*G*_bind_ = −37.83 kcal/mol) (Table 2) and other Omicron variants. Using Eq. 2, we obtain Δ*G*_bind_^exp^ = −9.13, −9.27, and −8.96 kcal/mol for RBD (N440K)-ACE2, RBD (N460K)-ACE2, and RBD (T478K)-ACE2, respectively. Thus, the in-silico variants with single key mutations have the binding energy well above −12 kcal/mol. Using Table 2 and eq 2, we obtain Δ*G*_bind_^exp^ = −11.27 kcal/mol for BA.2 with double mutations N440K, T478K, which suggests that triple mutations N440K, N460K, T478K will not lead to Δ*G*_bind_^exp^ much below −12 kcal/mol. This assumption is based on experimental results obtained since the beginning of the pandemic, which show that the highest binding affinity corresponds to *K*_D_ ∼ nM (or Δ*G*_bind_ ∼ −12 kcal/mol).^44^

### Electrostatic Interaction Dominates Over van der Waals Interaction in the RBD-ACE2 Complexes

Interchain electrostatic interactions are stronger than the van der Waals interactions in all subvariants (Table 2), which is similar to previous computational studies of the ancestral strain of SARS-CoV-2 and SARS-CoV-1.^12^ This makes sense since the RBD of the subvariants remains positively charged, while ACE2 is negatively charged. The dominance of electrostatics over van der Waals can lead to the idea that the stronger the electrostatic interaction, the lower the binding energy. However, this is not always the case as the BA.2 and BA4/5 lineages do not have improved electrostatic interaction compared to BA.1, but still have higher binding affinity (Table 2). This seems to contradict previous results of Omicron BA.1 binding compared to WT, as BA.1 improves electrostatic interaction to enhance affinity to ACE2.^35^ To resolve this controversy, we hypothesize that the emergence of Omicron will result in the advancement of antibodies which take advantage of electrostatic characteristics of Omicron BA.1 RBD, as the more negatively charged antibodies show improved affinity for RBD.^65^ Because the Omicron BA.1 RBD (+6e) is more positively charged than WT (+3e) and ACE2 is negatively charged, further improvement in electrostatic interaction with ACE2 may result in a worse ability to evade new antibodies. Thus, the virus evolves various ways to increase the binding affinity of the receptor but does not improve electrostatic interaction with ACE2. Although the electrostatic interaction improves in the novel lineages, the polar solvation energy is optimized to lower binding free energy (Table 2), which shows that the evolution of the virus improves other binding free energy terms, but does not focus only on the electrostatic interaction between RBD and ACE2.

Williams and Zhan proposed an elegant method to estimate binding free energy based on linear regression of polar solvation values.^66,67^ Guided by this idea, we have obtained the fit between the polar solvation term and the experimental binding free energy (Figure S3), which is
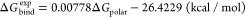
3

The correlation can be considered high,^68^ since the correlation coefficient *R* = 0.8. Thus, together with Williams and Zhan,^66,67^ our result indicates that the polar solvation energy could be used as an indicator of the binding affinity for ACE2 of various lineages.

### Contributions of Residues to Nonbonded Interaction between RBD and ACE2

**Figure 3:**
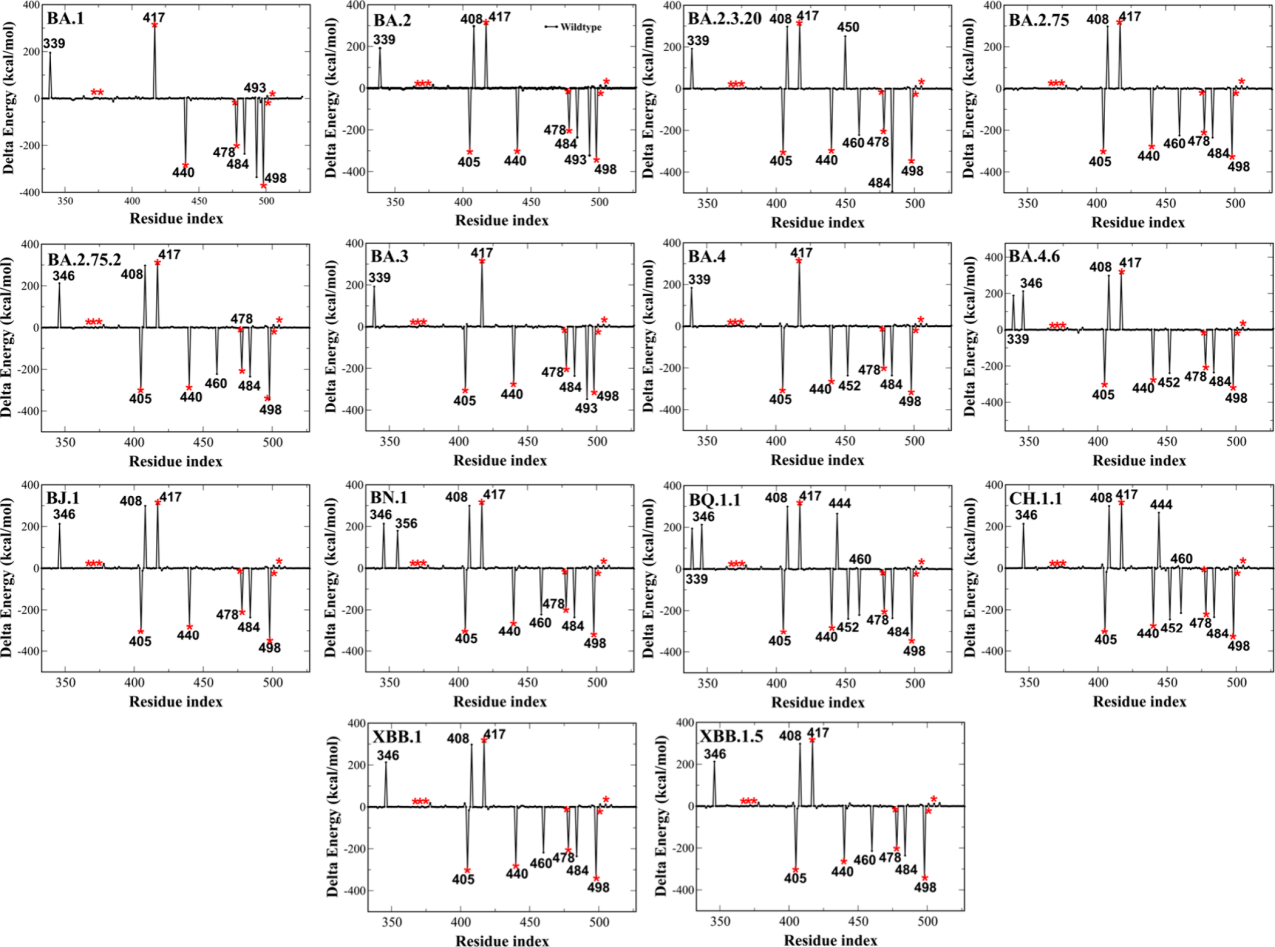
Change in nonbonded interaction energy of RBD residues between lineages and WT. Negative and positive values indicate improved and reduced interaction due to mutations. Data for BA.1 and WT are taken from ref (35). Red asterisks refer to mutations S371F, S373P, S375F, D405N, K417N, N440K, S477N, T478K, Q498R, N501Y, and Y505H which are present in all of the Omicron variants studied.

We calculated the nonbonded interaction between RBD and ACE2 residues, and then subtracted from these values the WT values obtained in our previous study.^35^ The result (Figure 3) shows the effect of mutations in these lineages on the RBD-ACE2 interaction. Mutations are concentrated in regions that have a side chain contact with ACE2 (Figure S4), which is logical, because the replacement of RBD residues at the border with ACE2 directly changes the interaction of these two proteins. The variants share many common mutations (Table S1) with mutations S371F, S373P, S375F, D405N, K417N, N440K, S477N, T478K, Q498R, N501Y, and Y505H present in all variants studied. The BA.1 variant does not have mutations S371F and D405N that appear in the remaining Omicron variants. At residue 339 there are two mutations G339D and G339H. The G339D mutation of RBD decreases the nonbonded interaction with ACE2 (Figure 3) in variants BA.1, BA.2, BA.2.3.20, BA.3, BA.4/BA.5, and BA.4.6, while the G339H mutation insignificantly changes the interaction. Triple mutations at 371, 373, and 375 trivially alter the RBD-ACE2 interaction. R346T and K356T in BA.2.75.2, BA.4.6, BJ.1, BN.1, BQ.1.1, CH.1.1, XBB.1, and XBB.1.5 decrease the binding affinity due to a reduced charge. The mutation D405N in all variants enhances the stability of the complex by replacing the negative D residue with a neutral N residue (Figure 3).

The R408S mutation in all variants except BA.1, BA.3, and BA.4/BA.5 weakens the interaction between RBD and ACE2 due to substitution of the positively charged R with the neutral residue S. K417N and N440K demonstrate contrasting contributions to the binding affinity. K417N decreases charge reducing nonbonded interaction, while N440K improves it. In experiments, it was found that the L452R mutation enhances the virus infectivity,^69,70^ which may be associated with increased interaction between RBD and ACE2 (Figure 3) in lineages BA.4, BA.4.6, BQ.1.1, and CH.1.1. The N460K mutation in BA.2.3.20, BA.2.75.2, BN.1, BQ.1.1, CH.1.1, XBB.1, and XBB.1.5 enhances the interaction by increasing the net charge, which is in line with experiments.^27,71^

Replacement of a neutral residue with another neutral residue as mutations V445P, G446S, S477N, F486S, F486V, F486P, F490V, F490S, S494P, N501Y, and Y505H has little effect on the nonbonded interaction (Figure 3). Mutations T478K, E484A, E484R, and Q498R enhance the interaction between RBD and ACE2, in particular, the E484R mutation substitutes the negatively charged residue by a positive charge residue in the BA.2.3.20 variant, which has a strong effect on the interaction energy.

**Figure 4:**
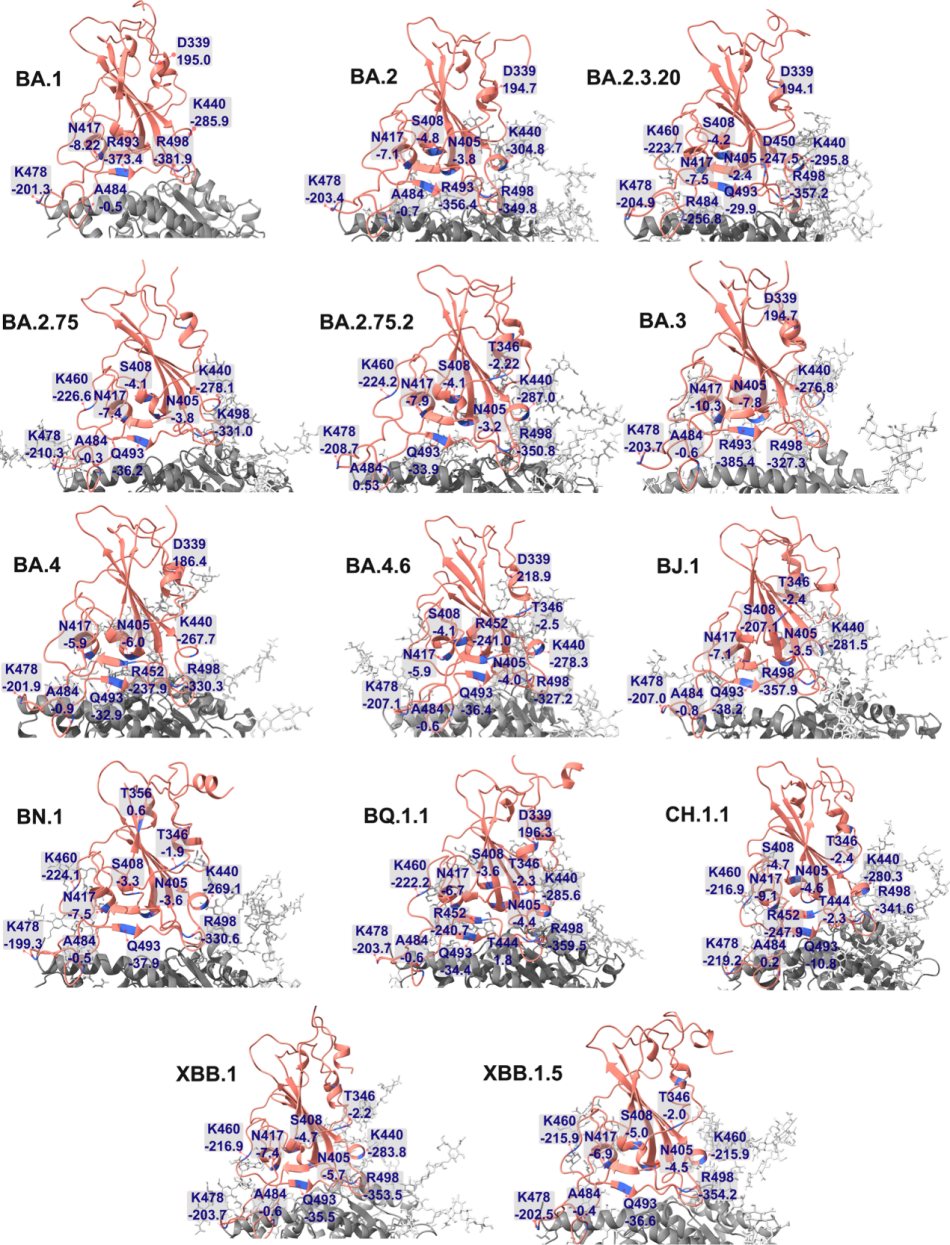
Residues that have a significant difference in nonbonded interaction with WT as shown in Figure 3. ACE2 is gray, ACE2 glycans are light gray, and RBD is salmon color. The interaction energy is indicated next to the residue.

Figure 4 shows residues that have a significant difference in nonbonded interaction energy with the WT (Figure 3). In many lineages, mutations R346T, R408S, and K417N, located near the interface between RBD and ACE2, attenuate the electrostatic interaction. Although these residues weaken the interaction between the two proteins, their contribution is still negative since the absolute value of their interaction energy is lower than that of the WT. The N residue at position 405 makes a negative contribution to the interaction, which may be due to the neutral charge of the N amino acid. Likewise, residue A at position 484 has a small negative nonbonded interaction. This is in contrast to mutation at 339 (G339D), in which replacing G with D increases the interaction energy by approximately 200 kcal/mol (see subvariants BA.1, BA.2, BA.2.3.20, BA.3, BA.4, BA.4.6, and BQ.1.1 in Figure 4). Near the contact surface, other mutations enhance the interaction, such as mutations N440K, L452R, N460K, T478K, and Q498R. These mutated residues have large positive charges, facilitating electrostatic interactions.

## Conclusions

As evident from Figure S5, the virus evolution during the last three years results in an increase in its binding affinity to ACE2, which corresponds to the decrease of Δ*G*_bind_ from about −10 to −12 kcal/mol. As mentioned above, based on experimental results obtained over the past four years, it can be speculated that the lower limit of binding free energy is Δ*G*_bind_ ∼ −12. kcal/mol. Consequently, further evolution does not lead to a significant change in the binding affinity of the virus to the ACE2. Our simulations of different Omicron variants also support this trend. However, the evolution of SARS-CoV-2 may lead to immune evasion, as shown by the experiment.^23,72^

Using molecular dynamics simulation and the MM-PBSA method, we obtained the binding free energies between the RBDs of the Omicron lineages and human ACE2. Binding free energies correlate well with the experimental data. The electrostatic interaction dominates over van der Waals. However, there are lineages that have a weaker electrostatic interaction than BA.1 but still have an improved binding free energy, indicating that SARS-CoV-2 finds a way to enhance receptor binding but does not need to improve the electrostatic interaction.

We found that mutations G339H, D405N, N440K, L452R, N460K, T478K, E484A, and Q498R improve RBD-ACE2 interaction. However, mutations G339D, R346T, K417N, R408S, and R493Q in several lineages weaken it. Our observations suggest the presence of hot spots in the RBD that support virulence predictions for future lineages.

Compared to XBB.1.5, the recent variant XBB.1.16 (March 2023) has only one distinct mutation at residue 478 in RBD (T478K in XBB.1.5 and T478R in XBB.1.16). Since K and R have the same +1 charge, we expect that the binding affinity obtained in this work for XBB.1.5 is also applicable for XBB.1.16.

Finally, it would be interesting to study the effect of ACE2 mutations on binding of SARS-CoV-2 to host cells. Work in this direction is underway.

## Supporting Information Available

Table S1: Mutations in RBD of lineages studied in this work. Table S2: Student’s *t* test *p*-values between two sets of binding free energies obtained by MM/PBSA for the WT and Omicron subvariants. Table S3: Nonbonded interaction energies of K440, K460, and K478 with ACE2. Table S4: MM/PBSA results for Δ*G*_bind_ of RBD (N440K)-ACE2, RBD (N460K)-ACE2, and RBD (T478K)-ACE2. Figure S1: RMSD versus time for BA.2 and XBB.1.5. Figure S2: Number of pairs of Omicron subvariants with a *p*-value greater than 0.05 as a function of time. Figure S3: Correlation between experimental binding free energy Δ*G*_bind_^exp^ and polar solvation energy obtained by MM-PBSA. The correlation coefficient *R* = 0.8. Figure S4: Population of RBD residues which form side chain contact with ACE2. Figure S5: Time dependence of the experimental binding free energy for different variants, including wild, Alpha, Beta, Gamma, Delta, and Omicron (PDF)
