# Supplementary material for: SARS-CoV-2 Omicron Subvariants Do Not Differ Much in Binding Affinity to Human ACE2: A Molecular Dynamics Study
Source: J Phys Chem B. 2024 Apr 2;128(14):3340–9. doi: 10.1021/acs.jpcb.3c06270 (PMC11017248; doi:10.1021/acs.jpcb.3c06270)
Supplement: Supplementary file 1 — jp3c06270_si_001.pdf [file jp3c06270_si_001.pdf]

SARS-CoV-2 Omicron Subvariants do not Differ Much in Binding Affinity to Human ACE2: A Molecular Dynamics Study

Hoang Linh Nguyen[1,2,*], Nguyen Quoc Thai[3,4,*], and Mai Suan Li[5,*]

[1]Institute of Fundamental and Applied Sciences, Duy Tan University, Ho Chi Minh City 700000, Vietnam

[2]Faculty of Environmental and Natural Sciences, Duy Tan University, Da Nang 550000, Vietnam

[3]Faculty of Physics, VNU University of Science, Vietnam National University, 334 Nguyen Trai, Hanoi 100000, Vietnam

[4]Dong Thap University, 783 Pham Huu Lau Street, Ward 6, Cao Lanh City, Dong Thap, Vietnam

[5]Institute of Physics, Polish Academy of Sciences, al. Lotnikow 32/46, 02-668, Warsaw, Poland

*Email: nguyenhoanglinh9@duytan.edu.vn, nqthai@dthu.edu.vn, masli@ifpan.edu.pl

Supporting Information

Table S1: Mutations in RBD of lineages studied in this work. Data are taken from cov-spectrum.org website[1]. The mutation positions that exist in many variants are highlighted.

	Lineage	Mutations
1	BA.2	G339D, S371F, S373P, S375F, T376A, D405N, R408S, K417N, **N440K**, S477N, T478K, E484A, Q493R, Q498R, N501Y, Y505H
2	BA.2.3.20	G339D, S371F, S373P, S375F, T376A, D405N, R408S, K417N, **N440K**, K444R, N450D, L452M, N460K, S477N, T478K, E484R, Q498R, N501Y, Y505H
3	BA.2.75	G339H, S371F, S373P, S375F, T376A, D405N, R408S, K417N, **N440K**, G446S, N460K, S477N, T478K, E484A, Q498R, N501Y, Y505H
4	BA.2.75.2	G339H, R346T, S371F, S373P, S375F, T376A, D405N, R408S, K417N, **N440K**, G446S, N460K, S477N, T478K, E484A, F486S, Q498R, N501Y, Y505H
5	BA.3	G339D, S371F, S373P, S375F, D405N, K417N, **N440K**, G446S, S477N, T478K, E484A, Q493R, Q498R, N501Y, Y505H
6	BA.4/BA.5	G339D, S371F, S373P, S375F, T376A, D405N, R408S, K417N, **N440K**, L452R, S477N, T478K, E484A, F486V, Q498R, N501Y, Y505H
7	BA.4.6	G339D, R346T, S371F, S373P, S375F, T376A, D405N, R408S, K417N, **N440K**, L452R, S477N, T478K, E484A, F486V, Q498R, N501Y, Y505H
8	BJ.1	G339H, R346T, L368I, S371F, S373P, S375F, T376A, D405N, R408S, K417N, **N440K**, V445P, G446S, S477N, T478K, V483A, E484A, F490V, Q493R, Q498R, N501Y, Y505H
9	BN.1	G339H, R346T, K356T, S371F, S373P, S375F, T376A, D405N, R408S, K417N, **N440K**, G446S, N460K, S477N, T478K, E484A, F490S, S494P, Q498R, N501Y, Y505H
10	BQ.1.1	G339D, R346T, S371F, S373P, S375F, T376A, D405N, R408S, K417N, **N440K**, K444T, G446S, N460K, S477N, T478K, E484A, F486V, Q498R, N501Y, Y505H
11	CH.1.1	G339H, R346T, S371F, S373P, S375F, T376A, D405N, R408S, K417N, **N440K**, K444T, G446S, L452R, N460K, S477N, T478K, E484A, F486S, Q498R, N501Y, Y505H
12	XBB/XBB.1	G339H, R346T, L368I, S371F, S373P, S375F, T376A, D405N, R408S, K417N, **N440K**, V445P, G446S, N460K, S477N, T478K, E484A, F486S, F490S, Q498R, N501Y, Y505H
13	XBB.1.5	G339H, R346T, L368I, S371F, S373P, S375F, T376A, D405N, R408S, K417N, **N440K**, V445P, G446S, N460K, S477N, T478K, E484A, F486P, F490S, Q498R, N501Y, Y505H

Table S2: Student's T-test *p*-values between two sets of binding free energies obtained by MM/PBSA for the WT and Omicron subvariants. Pairs with *p*-values > 0.05 are highlighted in gray. There are 91 pairs of omicron subvariants, and 59 of them (64%) have a *p*-value > 0.05.

	WT	BA.1	BA.2	BA.2.3.20	B.2.75	BA.2.75.2	BA.3	BA.4	BA.4.6	BJ.1	BN.1	BQ.1.1	CH.1.1	XBB	XBB.1.5
WT		0.0006	<0.0001	<0.0001	<0.0001	<0.0001	<0.0001	<0.0001	<0.0001	<0.0001	<0.0001	<0.0001	<0.0001	<0.0001	<0.0001
BA.1	0.0006		0.0303	0.0015	0.0001	0.0006	0.786	0.7866	0.0641	0.0377	0.0353	0.0016	0.0002	0.0048	0.0020
BA.2	<0.0001	0.0303		0.2169	0.0051	0.0168	0.0462	0.0904	0.1179	0.053	0.0296	0.0703	0.0458	0.0961	0.0537
BA.2.3.20	<0.0001	0.0015	0.2169		0.0019	0.0282	<0.0001	0.2729	0.4039	0.1101	0.0624	0.1921	0.0358	0.2542	0.1330
BA.2.75	<0.0001	0.0001	0.0051	0.0019		0.9528	<0.0001	0.0638	0.0203	0.0428	0.7377	0.1585	0.0070	0.3852	0.4567
BA.2.75.2	<0.0001	0.0006	0.0168	0.0282	0.9528		0.0002	0.1688	0.0917	0.1676	0.8183	0.3386	0.0991	0.4798	0.5669
BA.3	<0.0001	0.7866	0.0462	<0.0001	<0.0001	0.0002		0.0003	0.0002	<0.0001	0.0005	0.0005	<0.0001	0.0029	0.0010
BA.4	<0.0001	0.7866	0.0904	0.2729	0.0638	0.1688	0.0003		0.7199	0.8551	0.2801	0.7526	1.0000	0.6618	0.4801
BA.4.6	<0.0001	0.0641	0.1179	0.4039	0.0203	0.0917	0.0002	0.7199		0.5271	0.1697	0.5111	0.5783	0.4900	0.3191
BJ.1	<0.0001	0.0377	0.053	0.1101	0.0428	0.1676	<0.0001	0.8551	0.5271		0.2942	0.8469	0.7572	0.7259	0.5235
BN.1	<0.0001	0.0353	0.0296	0.0624	0.7377	0.8183	0.0005	0.2801	0.1697	0.2942		0.4310	0.2023	0.6260	0.7376
BQ.1.1	<0.0001	0.0016	0.0703	0.1921	0.1585	0.3386	0.0005	0.7526	0.5111	0.8469	0.4310		0.6824	0.8504	0.6733
CH.1.1	<0.0001	0.0002	0.0458	0.0358	0.0070	0.0991	<0.0001	1.0000	0.5783	0.7572	0.2023	0.6824		0.6162	0.4026
XBB	<0.0001	0.0048	0.0961	0.2542	0.3852	0.4798	0.0029	0.6618	0.4900	0.7259	0.6260	0.8504	0.6162		0.8578
XBB.1.5	<0.0001	0.002	0.0537	0.1330	0.4567	0.5669	0.0010	0.4801	0.3191	0.5235	0.7376	0.6733	0.4026	0.8578	
Number of pairs have p > 0.05	0	3	7	8	5	9	1	12	11	10	10	11	8	11	11

Table S3: Non-bonded interaction energies (kcal/mol) of K440, K460, and K478 with ACE2 in two cases: single mutants and when they are in BA.2 variant (N440K, T478K) and in XBB.1.5 (N460K). Results were obtained by the MM/PBSA method.

K at different positions	In single mutant	In variants BA.2 and XBB.1.5
K440	-283.65 ± 14.17	-306.16 ± 8.97
K460	-188.15 ± 12.11	-215.89 ± 6.47
K478	-182.85 ± 10.17	-203.99 ± 9.16

Table S4: MM/PBSA results for ΔG_{bind} (kcal/mol) of RBD (N440K)-ACE2, RBD (N460K)-ACE2, and RBD (T478K)-ACE2.

System	ΔE_{elec}	ΔE_{vdW}	ΔG_{polar}	$\Delta G_{nonpolar}$	$-T\Delta S$	ΔG_{bind}
N440K	-1138.67 ± 13.33	-148.67 ± 2.12	1231.82 ± 5.29	-24.37 ± 2.47	58.94 ± 3.29	-20.95 ± 3.12
N460K	-1062.82 ± 7.73	-144.40 ± 1.63	1148.43 ± 7.05	-22.29 ± 1.31	59.30 ± 2.62	-21.78 ± 3.79
T478K	-810.31 ± 5.37	-126.45 ± 2.89	899.45 ± 19.66	-29.70 ± 1.57	47.05 ± 3.18	-19.96 ± 3.27

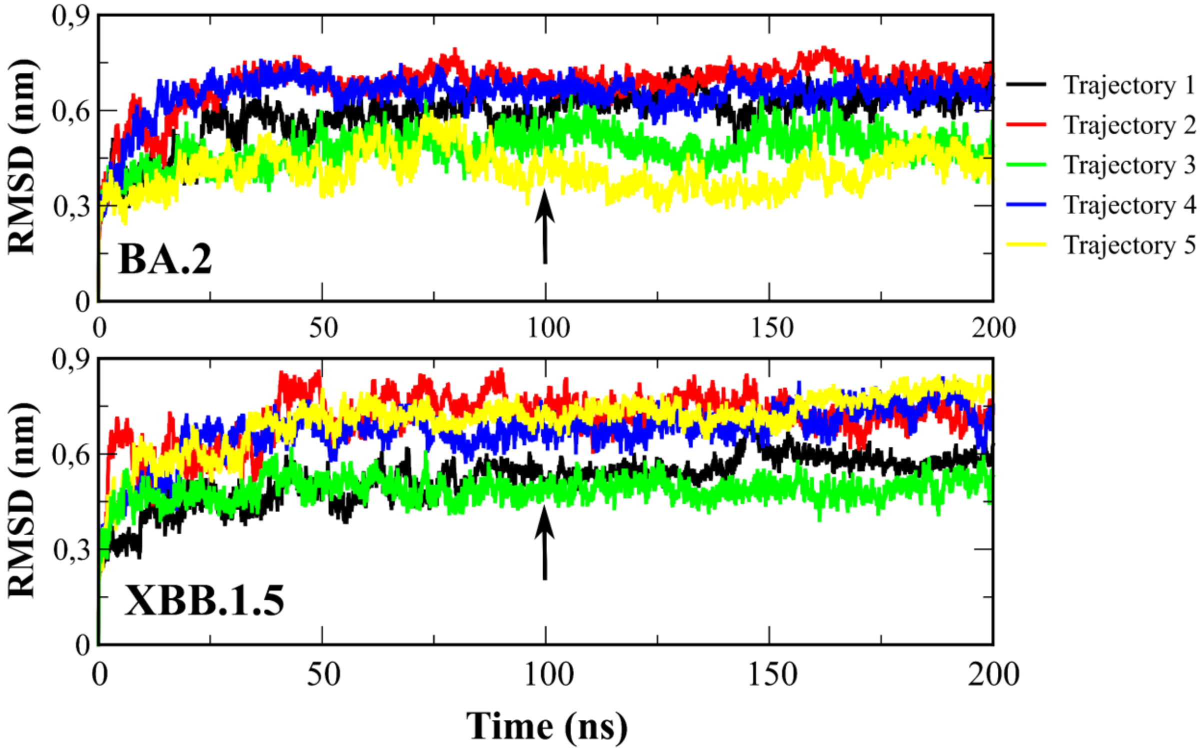

Figure S1: RMSD versus time for BA.2 and XBB.1.5. Arrow refers to 100 ns.

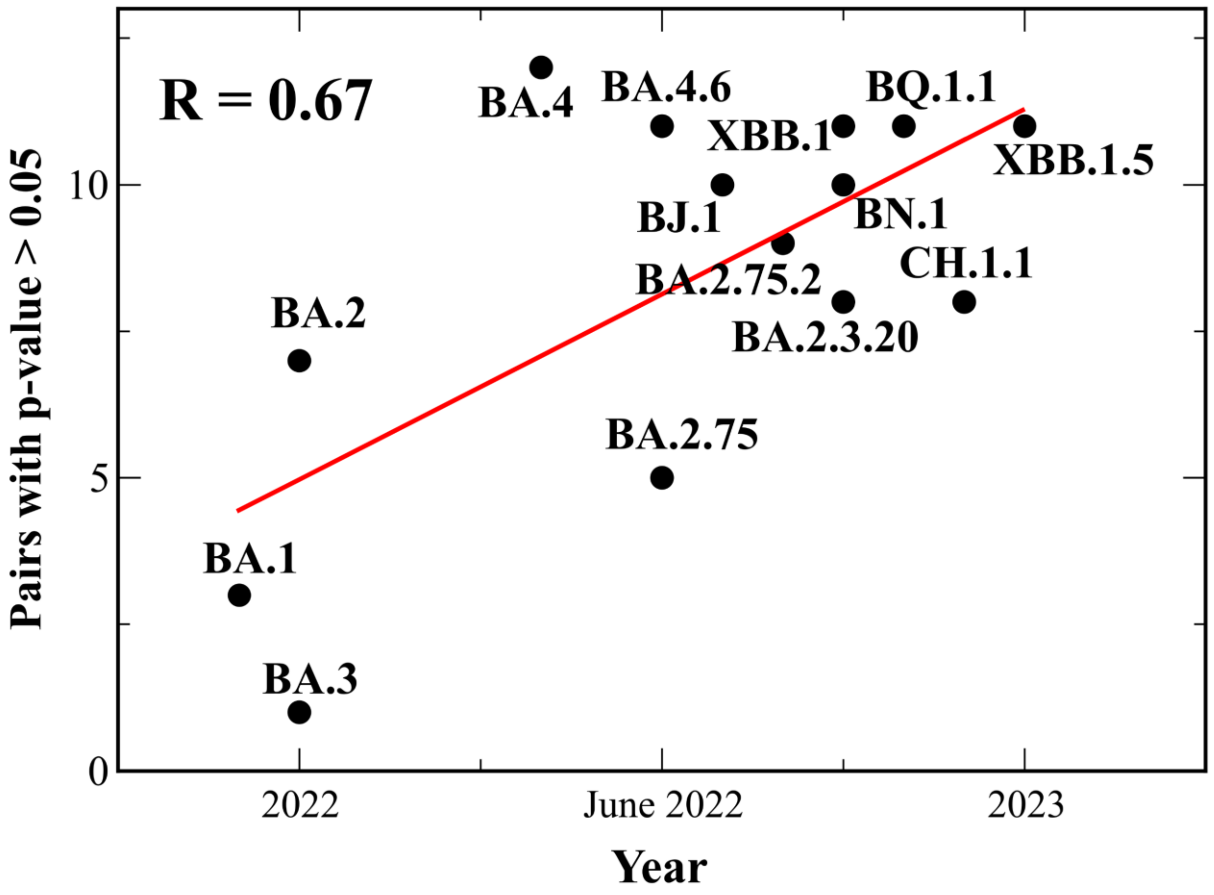

Figure S2. Number of pairs of Omicron subvariants with a *p*-value greater than 0.05 as a function of time. The red line is a linear fit y= (6.32 ± 2.02)* x - (12771.00 ± 4094.80) with correlation coefficient R = 0.67 ± 0.17.

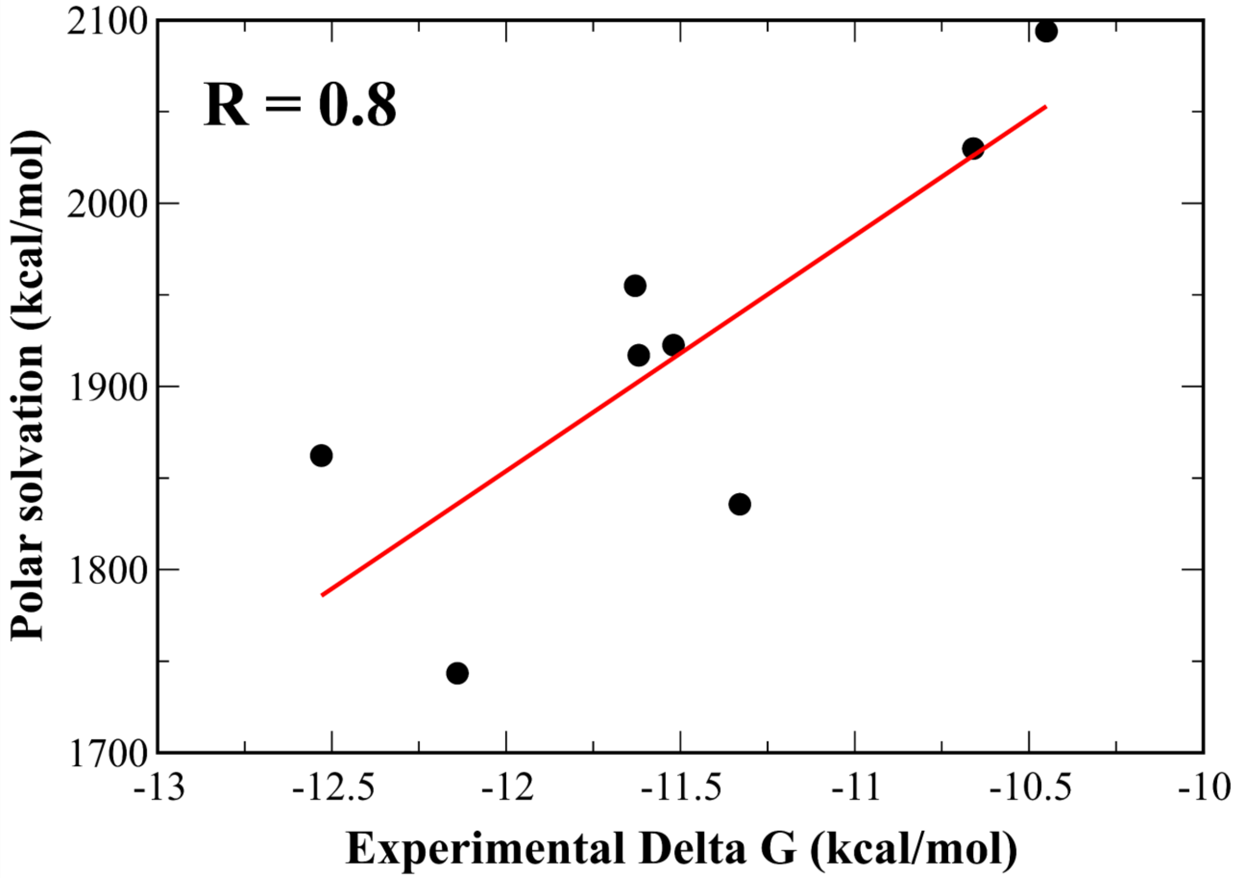

Figure S3: Correlation between experimental binding free energy ΔG_{bind}^{exp} and polar solvation energy obtained by MM-PBSA. The correlation coefficient *R* = 0.8.

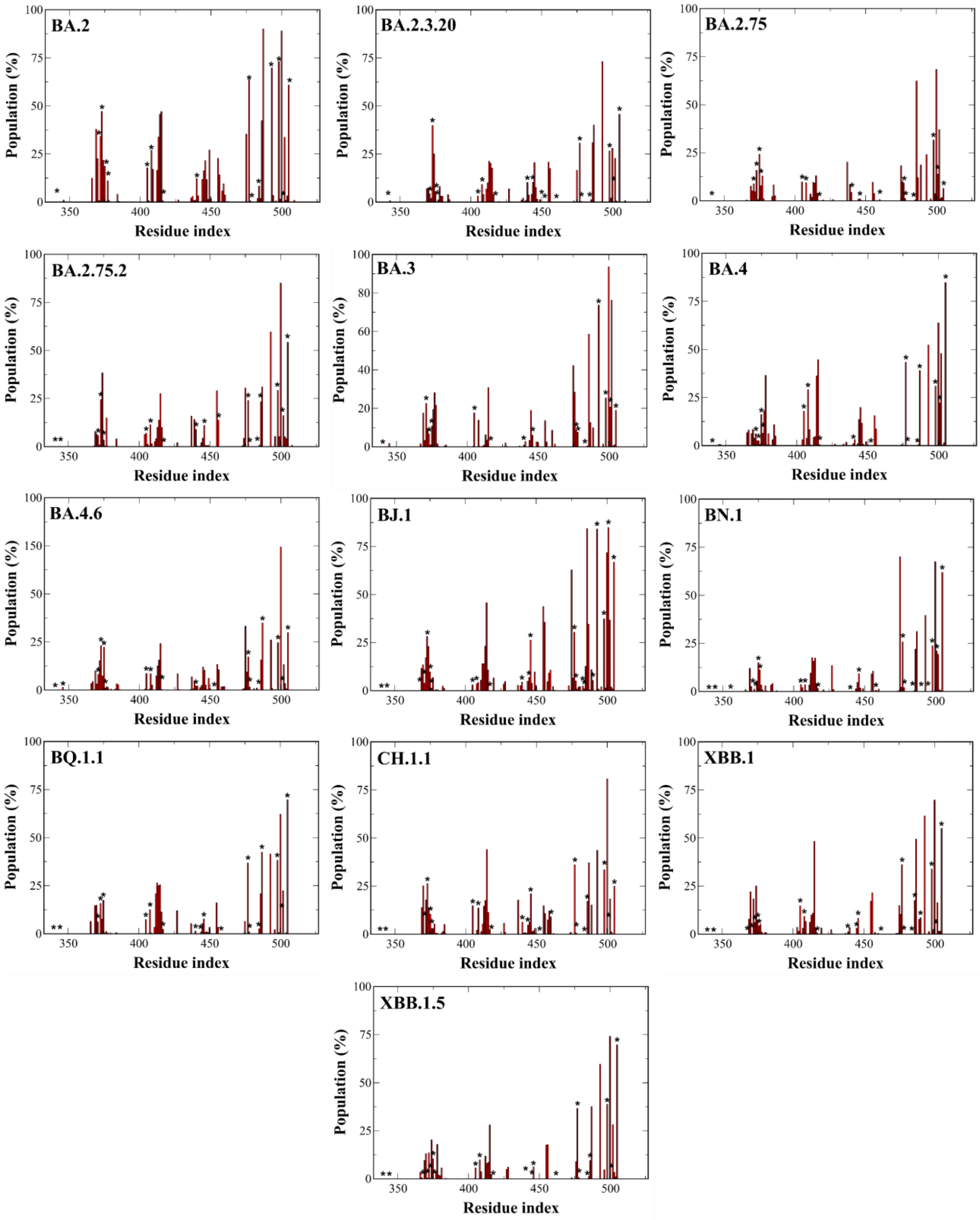

Figure S4: Population of RBD residues which form sidechain contact with ACE2. Mutation positions are marked with ★ symbol.

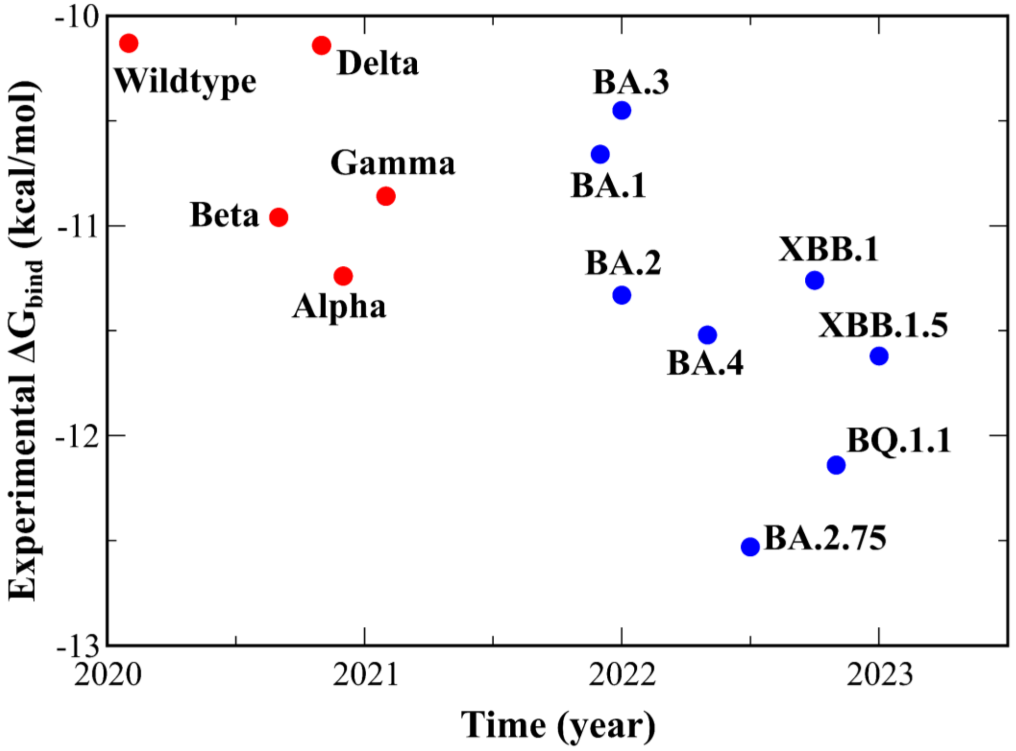

Figure S5: Time dependence of the experimental binding free energy for different variants, including Wildtype[2-19], Alpha[9-10,12,14-18,20], Beta[9-10,12-13,15,18,20], Gamma[9,12-13,18,20-22], Delta[9,16,20-21,23] and Omicron (blue ball refers to Omicron sub-lineages). The references to data for Omicron subvariants are listed in Table 1.

References

1. Chen, C.; Nadeau, S.; Yared, M.; Voinov, P.; Xie, N.; Roemer, C.; Stadler, T., CoV-Spectrum: analysis of globally shared SARS-CoV-2 data to identify and characterize new variants. *Bioinformatics* **2022,** *38* (6), 1735-1737.
2. Wrapp, D.; Wang, N.; Corbett, K. S.; Goldsmith, J. A.; Hsieh, C. L.; Abiona, O.; Graham, B. S.; McLellan, J. S., Cryo-EM structure of the 2019-nCoV spike in the prefusion conformation. *Science* **2020,** *367* (6483), 1260-1263.
3. Liu, H.; Zhang, Q.; Wei, P.; Chen, Z.; Aviszus, K.; Yang, J.; Downing, W.; Jiang, C.; Liang, B.; Reynoso, L.; Downey, G. P.; Frankel, S. K.; Kappler, J.; Marrack, P.; Zhang, G., The basis of a more contagious 501Y.V1 variant of SARS-CoV-2. *Cell Res* **2021,** *31* (6), 720-722.
4. Shang, J.; Wan, Y.; Luo, C.; Ye, G.; Geng, Q.; Auerbach, A.; Li, F., Cell entry mechanisms of SARS-CoV-2. *Proc Natl Acad Sci U S A* **2020,** *117* (21), 11727-11734.
5. Supasa, P.; Zhou, D.; Dejnirattisai, W.; Liu, C.; Mentzer, A. J.; Ginn, H. M.; Zhao, Y.; Duyvesteyn, H. M. E.; Nutalai, R.; Tuekprakhon, A.; Wang, B.; Paesen, G. C.; Slon-Campos, J.; Lopez-Camacho, C.; Hallis, B.; Coombes, N.; Bewley, K. R.; Charlton, S.; Walter, T. S.; Barnes, E.; Dunachie, S. J.; Skelly, D.; Lumley, S. F.; Baker, N.; Shaik, I.; Humphries, H. E.; Godwin, K.; Gent, N.; Sienkiewicz, A.; Dold, C.; Levin, R.; Dong, T.; Pollard, A. J.; Knight, J. C.; Klenerman, P.; Crook, D.;

Lambe, T.; Clutterbuck, E.; Bibi, S.; Flaxman, A.; Bittaye, M.; Belij-Rammerstorfer, S.; Gilbert, S.; Hall, D. R.; Williams, M. A.; Paterson, N. G.; James, W.; Carroll, M. W.; Fry, E. E.; Mongkolsapaya, J.; Ren, J.; Stuart, D. I.; Screaton, G. R., Reduced neutralization of SARS-CoV-2 B.1.1.7 variant by convalescent and vaccine sera. *Cell* **2021,** *184* (8), 2201-2211 e7.
6. Cameroni, E.; Bowen, J. E.; Rosen, L. E.; Saliba, C.; Zepeda, S. K.; Culap, K.; Pinto, D.; VanBlargan, L. A.; De Marco, A.; di Iulio, J.; Zatta, F.; Kaiser, H.; Noack, J.; Farhat, N.; Czudnochowski, N.; Havenar-Daughton, C.; Sprouse, K. R.; Dillen, J. R.; Powell, A. E.; Chen, A.; Maher, C.; Yin, L.; Sun, D.; Soriaga, L.; Bassi, J.; Silacci-Fregni, C.; Gustafsson, C.; Franko, N. M.; Logue, J.; Iqbal, N. T.; Mazzitelli, I.; Geffner, J.; Grifantini, R.; Chu, H.; Gori, A.; Riva, A.; Giannini, O.; Ceschi, A.; Ferrari, P.; Cippa, P. E.; Franzetti-Pellanda, A.; Garzoni, C.; Halfmann, P. J.; Kawaoka, Y.; Hebner, C.; Purcell, L. A.; Piccoli, L.; Pizzuto, M. S.; Walls, A. C.; Diamond, M. S.; Telenti, A.; Virgin, H. W.; Lanzavecchia, A.; Snell, G.; Veesler, D.; Corti, D., Broadly neutralizing antibodies overcome SARS-CoV-2 Omicron antigenic shift. *Nature* **2022,** *602* (7898), 664-670.
7. Zhang, X.; Wu, S.; Wu, B.; Yang, Q.; Chen, A.; Li, Y.; Zhang, Y.; Pan, T.; Zhang, H.; He, X., SARS-CoV-2 Omicron strain exhibits potent capabilities for immune evasion and viral entrance. *Signal Transduct Target Ther* **2021,** *6* (1), 430.
8. Chan, K. K.; Dorosky, D.; Sharma, P.; Abbasi, S. A.; Dye, J. M.; Kranz, D. M.; Herbert, A. S.; Procko, E., Engineering human ACE2 to optimize binding to the spike protein of SARS coronavirus 2. *Science* **2020,** *369* (6508), 1261-1265.
9. Dejnirattisai, W.; Huo, J.; Zhou, D.; Zahradnik, J.; Supasa, P.; Liu, C.; Duyvesteyn, H. M. E.; Ginn, H. M.; Mentzer, A. J.; Tuekprakhon, A.; Nutalai, R.; Wang, B.; Dijokaite, A.; Khan, S.; Avinoam, O.; Bahar, M.; Skelly, D.; Adele, S.; Johnson, S. A.; Amini, A.; Ritter, T. G.; Mason, C.; Dold, C.; Pan, D.; Assadi, S.; Bellass, A.; Omo-Dare, N.; Koeckerling, D.; Flaxman, A.; Jenkin, D.; Aley, P. K.; Voysey, M.; Costa Clemens, S. A.; Naveca, F. G.; Nascimento, V.; Nascimento, F.; Fernandes da Costa, C.; Resende, P. C.; Pauvolid-Correa, A.; Siqueira, M. M.; Baillie, V.; Serafin, N.; Kwatra, G.; Da Silva, K.; Madhi, S. A.; Nunes, M. C.; Malik, T.; Openshaw, P. J. M.; Baillie, J. K.; Semple, M. G.; Townsend, A. R.; Huang, K. A.; Tan, T. K.; Carroll, M. W.; Klenerman, P.; Barnes, E.; Dunachie, S. J.; Constantinides, B.; Webster, H.; Crook, D.; Pollard, A. J.; Lambe, T.; Consortium, O.; Consortium, I. C.; Paterson, N. G.; Williams, M. A.; Hall, D. R.; Fry, E. E.; Mongkolsapaya, J.; Ren, J.; Schreiber, G.; Stuart, D. I.; Screaton, G. R., SARS-CoV-2 Omicron-B.1.1.529 leads to widespread escape from neutralizing antibody responses. *Cell* **2022,** *185* (3), 467-484 e15.
10. Tian, F.; Tong, B.; Sun, L.; Shi, S.; Zheng, B.; Wang, Z.; Dong, X.; Zheng, P., N501Y mutation of spike protein in SARS-CoV-2 strengthens its binding to receptor ACE2. *Elife* **2021,** *10*.
11. Yin, W.; Xu, Y.; Xu, P.; Cao, X.; Wu, C.; Gu, C.; He, X.; Wang, X.; Huang, S.; Yuan, Q.; Wu, K.; Hu, W.; Huang, Z.; Liu, J.; Wang, Z.; Jia, F.; Xia, K.; Liu, P.; Wang, X.; Song, B.; Zheng, J.; Jiang, H.; Cheng, X.; Jiang, Y.; Deng, S. J.; Xu, H. E., Structures of the Omicron spike trimer with ACE2 and an anti-Omicron antibody. *Science* **2022,** *375* (6584), 1048-1053.
12. Han, P.; Su, C.; Zhang, Y.; Bai, C.; Zheng, A.; Qiao, C.; Wang, Q.; Niu, S.; Chen, Q.; Zhang, Y.; Li, W.; Liao, H.; Li, J.; Zhang, Z.; Cho, H.; Yang, M.; Rong, X.; Hu, Y.; Huang, N.; Yan, J.; Wang, Q.; Zhao, X.; Gao, G. F.; Qi, J., Molecular insights into receptor binding of recent emerging SARS-CoV-2 variants. *Nat Commun* **2021,** *12* (1), 6103.
13. Barton, M. I.; MacGowan, S. A.; Kutuzov, M. A.; Dushek, O.; Barton, G. J.; van der Merwe, P. A., Effects of common mutations in the SARS-CoV-2 Spike RBD and its ligand, the human ACE2 receptor on binding affinity and kinetics. *Elife* **2021,** *10*.
14. Collier, D. A.; De Marco, A.; Ferreira, I.; Meng, B.; Datir, R. P.; Walls, A. C.; Kemp, S. A.; Bassi, J.; Pinto, D.; Silacci-Fregni, C.; Bianchi, S.; Tortorici, M. A.; Bowen, J.; Culap, K.; Jaconi, S.; Cameroni, E.; Snell, G.; Pizzuto, M. S.; Pellanda, A. F.; Garzoni, C.; Riva, A.; Collaboration, C.-N. B. C.-.; Elmer, A.; Kingston, N.; Graves, B.; McCoy, L. E.; Smith, K. G. C.; Bradley, J. R.; Temperton, N.; Ceron-Gutierrez, L.; Barcenas-Morales, G.; Consortium, C.-G. U.; Harvey, W.; Virgin, H. W.; Lanzavecchia, A.; Piccoli, L.; Doffinger, R.; Wills, M.; Veesler, D.; Corti, D.; Gupta, R. K., Sensitivity of SARS-CoV-2 B.1.1.7 to mRNA vaccine-elicited antibodies. *Nature* **2021,** *593* (7857), 136-141.

15. Laffeber, C.; de Koning, K.; Kanaar, R.; Lebbink, J. H. G., Experimental Evidence for Enhanced Receptor Binding by Rapidly Spreading SARS-CoV-2 Variants. *J Mol Biol* **2021,** *433* (15), 167058.
16. McCallum, M.; Walls, A. C.; Sprouse, K. R.; Bowen, J. E.; Rosen, L. E.; Dang, H. V.; De Marco, A.; Franko, N.; Tilles, S. W.; Logue, J.; Miranda, M. C.; Ahlrichs, M.; Carter, L.; Snell, G.; Pizzuto, M. S.; Chu, H. Y.; Van Voorhis, W. C.; Corti, D.; Veesler, D., Molecular basis of immune evasion by the Delta and Kappa SARS-CoV-2 variants. *Science* **2021,** *374* (6575), 1621-1626.
17. Bayarri-Olmos, R.; Johnsen, L. B.; Idorn, M.; Reinert, L. S.; Rosbjerg, A.; Vang, S.; Hansen, C. B.; Helgstrand, C.; Bjelke, J. R.; Bak-Thomsen, T.; Paludan, S. R.; Garred, P.; Skjoedt, M. O., The alpha/B.1.1.7 SARS-CoV-2 variant exhibits significantly higher affinity for ACE-2 and requires lower inoculation doses to cause disease in K18-hACE2 mice. *Elife* **2021,** *10*.
18. Koehler, M.; Ray, A.; Moreira, R. A.; Juniku, B.; Poma, A. B.; Alsteens, D., Molecular insights into receptor binding energetics and neutralization of SARS-CoV-2 variants. *Nat Commun* **2021,** *12* (1), 6977.
19. Cui, Z.; Liu, P.; Wang, N.; Wang, L.; Fan, K.; Zhu, Q.; Wang, K.; Chen, R.; Feng, R.; Jia, Z.; Yang, M.; Xu, G.; Zhu, B.; Fu, W.; Chu, T.; Feng, L.; Wang, Y.; Pei, X.; Yang, P.; Xie, X. S.; Cao, L.; Cao, Y.; Wang, X., Structural and functional characterizations of infectivity and immune evasion of SARS-CoV-2 Omicron. *Cell* **2022,** *185* (5), 860-871 e13.
20. Cao, Y.; Song, W.; Wang, L.; Liu, P.; Yue, C.; Jian, F.; Yu, Y.; Yisimayi, A.; Wang, P.; Wang, Y.; Zhu, Q.; Deng, J.; Fu, W.; Yu, L.; Zhang, N.; Wang, J.; Xiao, T.; An, R.; Wang, J.; Liu, L.; Yang, S.; Niu, X.; Gu, Q.; Shao, F.; Hao, X.; Jin, R.; Wang, Y.; Xie, X. S.; Wang, X., **2022**.
21. Zhang, J.; Xiao, T.; Cai, Y.; Lavine, C. L.; Peng, H.; Zhu, H.; Anand, K.; Tong, P.; Gautam, A.; Mayer, M. L.; Walsh, R. M., Jr.; Rits-Volloch, S.; Wesemann, D. R.; Yang, W.; Seaman, M. S.; Lu, J.; Chen, B., Membrane fusion and immune evasion by the spike protein of SARS-CoV-2 Delta variant. *Science* **2021,** *374* (6573), 1353-1360.
22. Mannar, D.; Saville, J. W.; Sun, Z.; Zhu, X.; Marti, M. M.; Srivastava, S. S.; Berezuk, A. M.; Zhou, S.; Tuttle, K. S.; Sobolewski, M. D.; Kim, A.; Treat, B. R.; Da Silva Castanha, P. M.; Jacobs, J. L.; Barratt-Boyes, S. M.; Mellors, J. W.; Dimitrov, D. S.; Li, W.; Subramaniam, S., SARS-CoV-2 variants of concern: spike protein mutational analysis and epitope for broad neutralization. *Nat Commun* **2022,** *13* (1), 4696.
23. Saville, J. W.; Mannar, D.; Zhu, X.; Srivastava, S. S.; Berezuk, A. M.; Demers, J. P.; Zhou, S.; Tuttle, K. S.; Sekirov, I.; Kim, A.; Li, W.; Dimitrov, D. S.; Subramaniam, S., Structural and biochemical rationale for enhanced spike protein fitness in delta and kappa SARS-CoV-2 variants. *Nat Commun* **2022,** *13* (1), 742.
